# Letter in Response to “Reconsidering Case Reports: A Modest Call from a Seasoned Clinician”

**DOI:** 10.31662/jmaj.2025-0538

**Published:** 2026-03-19

**Authors:** Srujana Mohanty

**Affiliations:** 1 Department of Microbiology, All India Institute of Medical Sciences, Bhubaneswar, Odisha, India

**Keywords:** case, case report, concept, journal, original article

## To the Editor

I read with interest the article by Matsubara entitled “Reconsidering Case Reports: A Modest Call from a Seasoned Clinician” in the October 2025 issue of *JMA Journal* ^(1)^. The views of the author on the importance of case reports in medical literature truly drive home the point and have been expressed at an extremely opportune time, when as medical researchers we are encountering many emerging diseases because of either increasing awareness or being fortunate enough to be working with new cutting-edge technology and medical equipment enabling us to identify newer diseases. Somehow, a published report of a rare or uncommon clinical entity by oneself does unleash or trigger a thinking process to be vigilant for more cases, beckoning a deeper exploration of more such experiences leading to an original study.

I would like to share some of my own experiences, in my more than 25 years as a clinical microbiologist, to emphasize that case reports sow the seeds of imagination for bigger, more systematically planned research projects and are the stepping stones for a more fruitful scientific outcome. Some such examples include a single case report of *Enterococcus* species brain abscess being followed by more expansive work on characterization of clinical *Enterococcus* isolates ^(2), (3)^ and a single case study of melioidosis leading to a more complex work on localized melioidosis cases over a 2-year period ^(4), (5)^.

The importance of case reports in expanding our knowledge of the medical literature cannot be overemphasized because it alerts the clinician to subtle variations in the routine findings, triggering an interest in deeper exploration of the idea/concept in question, often yielding impactful outcomes. Efforts should be made to acknowledge the case report as an important scientific contribution, if not as alluring a publication option as an original article.

## Article Information

### Author Contributions

Srujana Mohanty designed the study, wrote, edited, and approved the final manuscript, and met the ICMJE criteria for authorship.

### Conflicts of Interest

None

### Ethics Approval

The All India Institute of Medical Sciences Bhubaneswar, Odisha, India does not demand institutional review board approval for this type of study. Patient anonymity preservation and informed consent for reporting are not applicable.

### Data Availability Statement

Data sharing is not applicable to this article because no new data were created or analyzed in this study.
